# Specificity and Efficiency of the Uracil DNA Glycosylase-Mediated Strand Cleavage Surveyed on Large Sequence Libraries

**DOI:** 10.1038/s41598-019-54044-x

**Published:** 2019-11-28

**Authors:** Kathrin Hölz, Angelina Pavlic, Jory Lietard, Mark M. Somoza

**Affiliations:** 1 Institute of Inorganic Chemistry, Faculty of Chemistry, University of Vienna, Vienna, Austria; 2 Chair of Food Chemistry and Molecular and Sensory Science, Technical University of Munich, Lise-Meitner-Straße 34, D-85354 Freising, Germany

**Keywords:** Nucleic acids, Combinatorial libraries, DNA nanotechnology, Biochemistry, DNA, Enzymes

## Abstract

Uracil-DNA glycosylase (UDG) is a critical DNA repair enzyme that is well conserved and ubiquitous in nearly all life forms. UDG protects genomic information integrity by catalyzing the excision from DNA of uracil nucleobases resulting from misincorporation or spontaneous cytosine deamination. UDG-mediated strand cleavage is also an important tool in molecular biotechnology, allowing for controlled and location-specific cleavage of single- and double DNA chemically or enzymatically synthesized with single or multiple incorporations of deoxyuridine. Although the cleavage mechanism is well-understood, detailed knowledge of efficiency and sequence specificity, in both single and double-stranded DNA contexts, has so far remained incomplete. Here we use an experimental approach based on the large-scale photolithographic synthesis of uracil-containing DNA oligonucleotides to comprehensively probe the context-dependent uracil excision efficiency of UDG.

## Introduction

**Figure 1:**
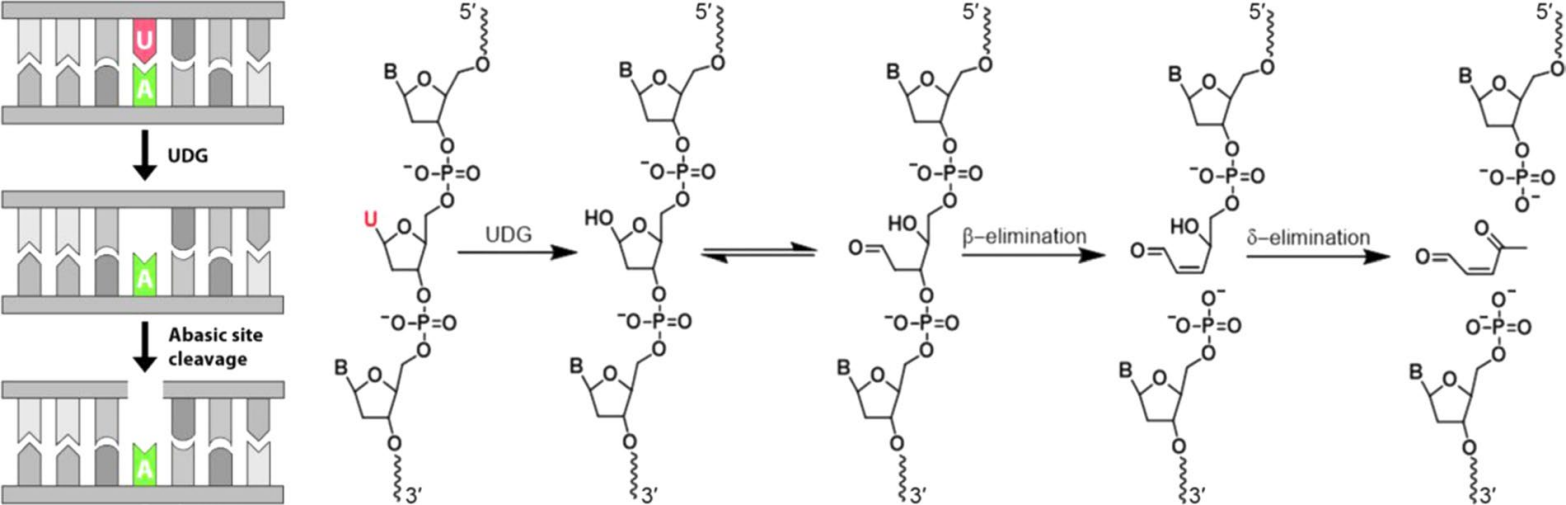
(Left) Schematic illustration of the UDG-mediated generation of single nucleotide gaps on nucleic acid strands. In a first step, UDG catalyzes the excision of uracil, leading to the formation of an abasic site. This AP-site can then either be cleaved by the lyase activity of specific endonucleases, or chemically. The USER enzyme, a mixture of UDG and Endonuclease VIII, combines AP-site formation and cleavage in a single solution. (Right) Molecular structures indicating the generation of a single nucleotide gap/strand cleavage via a β- and a subsequent δ-elimination reaction. First, UDG hydrolyzes the glycosidic bond from the uracil-containing DNA strand. The ribose at the apyrimidinic site lacks a glycosidic bond and is therefore highly unstable and converts rapidly into its reactive open-chain aldehyde, its hemiacetal or its hydrate form. The lyase activity of AP-endonucleases, or the exposure to either basic or acidic conditions, initiates a β-elimination reaction, resulting in the cleavage of the phosphodiester backbone 3′ to the AP-site and the formation of an α,β-unsaturated aldehyde. Subsequent δ-elimination induces DNA strand cleavage 5′ to the AP-site resulting in the generation of a single-nucleotide gap in dsDNA or strand cleavage in ssDNA.

Uracil-DNA glycosylase (UDG) is a highly conserved repair enzyme that catalyzes the excision of uracil from uracil-containing single- and double-stranded DNA^1–7^. Its main function is the prevention of mutagenesis that could occur due to misincorporation of uracil during DNA synthesis or spontaneous cytosine deamination in DNA strands^8,9^; it does so by initiating the base-excision repair (BER) pathway^10–12^. Combining processive and distributive mechanisms, UDG slides along the DNA strands, recognizes uracil molecules and proceeds to base excision by flipping the base into its active site pocket^13–15^. More precisely, UDG binds, kinks and compresses the DNA backbone (also called ‘pinch-push-pull’-mechanism^14,16–18^) to actually scan the minor groove in sections for damage^10^. Once it recognizes a uracil base, it actively flips the dU nucleotide into an extrahelical conformation^12,16^ and eliminates uracil from DNA by cleaving the *N*-glycosidic bond^14,16–18^ that results in the formation of an abasic site (AP-site). Such AP-sites do not only result from treatment with UDG, but can also form after chemical cleavage of the *N*-glycosidic bond or as intermediates in the repair of DNA lesions and, as such, represent one of the most common lesions in the DNA of eukaryotic cells^19^. Repairing AP-sites is essential since the remaining deoxyribose interconverts between hydrate, hemiacetal and the highly reactive open-chain aldehyde forms (Fig. 1) that have the potential to react with other cell components, leading to strand breakage or mutations^19–21^. *In vivo*, UDG remains bound to the AP-site until it is replaced by specific endonucleases with a much higher affinity to abasic sites^22^. Such enzymes, like e.g. Endonuclease VIII or Exonuclease III, have an AP-lyase activity that catalyzes the cleavage of the phosphodiester backbone 3′ and/or 5′ of the AP-site, releasing the base-free deoxyribose, and thus forming a single-nucleotide gap^23,24^.

While the removal of uracil by UDG and the associated initiation of the BER pathway is essential in eukaryotic cells, since it prevents the propagation of mutagenic threats through DNA replication^25^, its repair function can also be harnessed *in vitro* as an enzymatic approach for the base-specific cleavage of DNA oligonucleotides. A convenient way to enzymatically generate uracil nucleotide gaps *in vitro* is through the USER (Uracil-Specific Excision Reagent) enzyme—a mixture of the enzymes UDG and Endonuclease VIII—that can generate single-nucleotide gaps at the location of deoxyuridine residues. However, since abasic sites are very labile under various conditions, the cleavage of the phosphodiester backbone at the AP-site can also be induced non-enzymatically. The aldehyde form especially makes the AP-sites highly sensitive to cleavage under enzymatic as well as under high or low pH conditions^20^. Under alkaline conditions, the DNA at the AP-site is either cleaved in a β- or a β,δ-elimination reaction^26–29^. The presence of nucleophilic amines initiates a β-elimination reaction through deprotonation of the aldehyde at the α-position, leading to cleavage of the 3′-phosphodiester bond and to the formation of an α,β-unsaturated aldehyde. A subsequent deprotonation at the δ-position initiates cleavage of the 5′-phosphodiester bond, generating a single nucleotide gap as indicated in Fig. 1. Under acidic conditions or in presence of an organic base, a Schiff base is formed from a condensation reaction between amino nucleophiles and the aldehyde form. Subsequently, a β-elimination reaction takes place, leading to 3′-site DNA strand cleavage^20^. An alternative strategy to cleave abasic sites was proposed by Shishkina and Johnson, who induced dehydration of AP-sites by evaporating their probes to dryness^30^. Such chemical strategies to cleave abasic sites on DNA strands provide an alternative to Endonuclease VIII and allow for the kinetics of UDG processing to be determined independently of those of Endonuclease VIII. Well-understood and efficient cleavage methods that allow for a controlled release of DNA *in vitro* and from surfaces, such as microarrays, have become particularly relevant in the context of nucleic acid library preparation^31–34^ and RNA capture in spatial transcriptomics^35,36^.

Microarrays are valuable bioanalytical tools allowing for any given experiment to be simultaneously performed on all probes present on the surface^37,38^. Complex and high-density DNA arrays can be obtained using maskless array synthesis (MAS)^39^, where hundreds of thousands of unique nucleic acid sequences are synthesized *in situ* using UV light as a means to control the sequential polymerization of phosphoramidites. While microarrays were initially used for gene expression profiling^40,41^, their range of applications has since then significantly broadened to include, among others, genotyping^42^ and resequencing^43^. In general, microarray applications can be separated into three types: hybridization-based studies, enzymatic interactions and protein-binding experiments, or a combination of these two types. While hybridization allows for a direct visualization of the experimental outcome thanks to the use of fluorescent labels, enzyme assays open up the way to further processing that cannot be directly performed on the microarray surface, such as sequencing of the DNA strands. Alternatively, enzymatic cleavage may simply be a step within a more complex, multi-stage experimental setup involving microarrays as, for instance, in *de novo* gene assembly^31,32,44^, spatial expression profiling^35^ or the formation of DNA nanostructures^45^. At a more fundamental level however, high-density microarrays are an ideal platform for the study of enzyme kinetics and substrate preferences. With UDG-mediated degradation being routinely used in molecular biology and a potentially very useful technique for DNA library recovery off-array, addressing the issue of efficiency according to sequence context would help in designing optimal dU-containing substrates for enzymatic cleavage.

In this report, we wished to apply UDG-mediated degradation of DNA synthesized on high-density arrays, with the aim to develop a protocol for an efficient excision of dU nucleotides and, consequently, efficient oligonucleotide cleavage from the surface. To do so, we performed a series of studies on the UDG-mediated cleavage of uracil nucleotides on high-density DNA microarrays in combination with chemically-induced AP-site cleavage. We also focused our attention on studying the sequence dependence of UDG-mediated degradation of single- and double-stranded DNA. The results and the data collected was then further used to develop and optimize uracil cleavage sites.

## Results and Discussion

### UDG-mediated uracil excision and chemical AP site cleavage on microarrays

**Figure 2:**
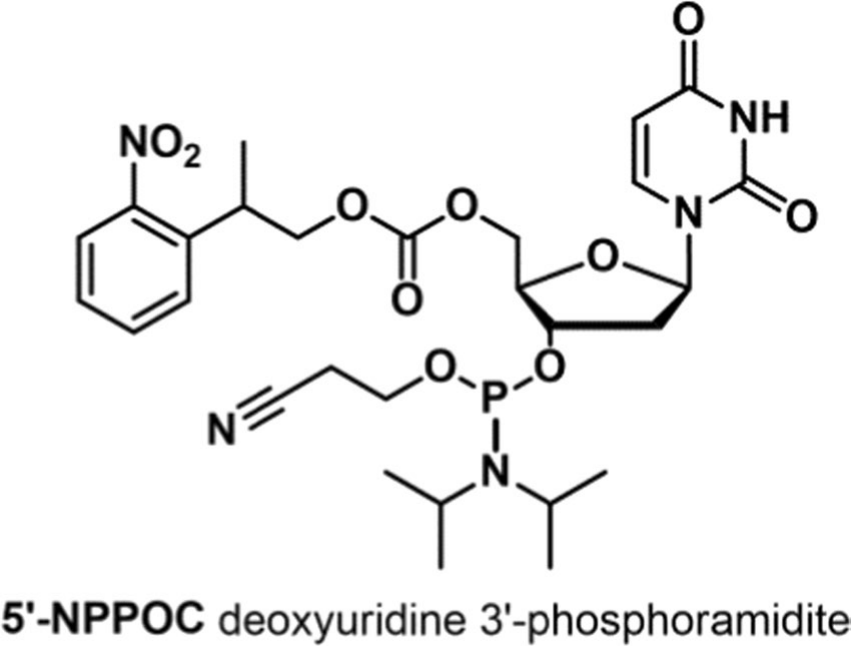
Molecular structure of the 5′-NPPOC deoxyuridine 3′-phosphoramidite (in the text referred to as dU or uracil nucleotide.

**Figure 3:**
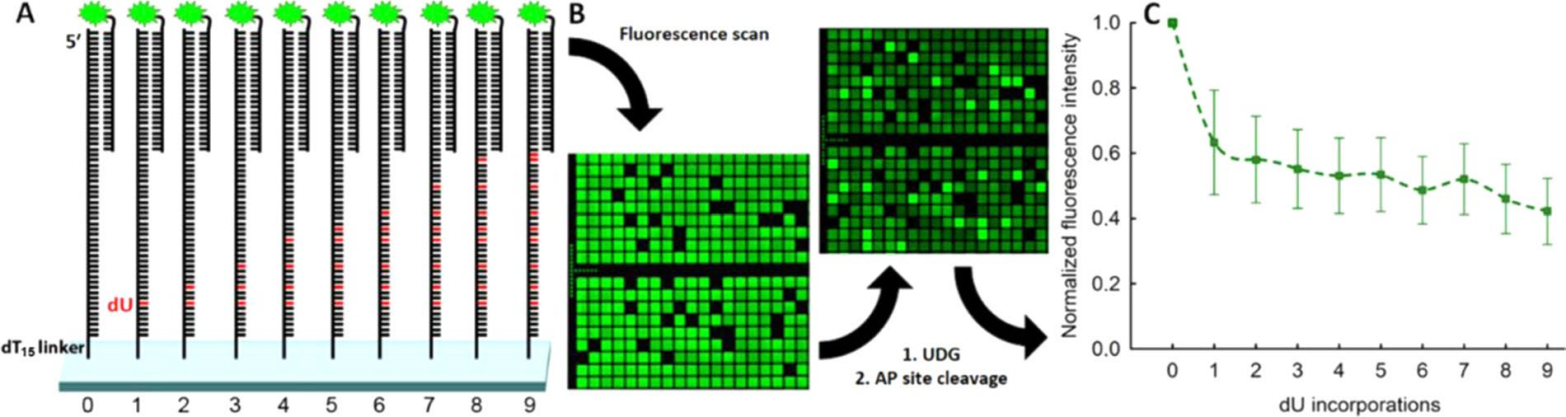
(**A**) Sequence design for the investigation of uracil and abasic site cleavage on microarrays. Each sequence consists of a dT_15_-linker, then a 30mer with either no dUs (control) or an increasing number of dU-incorporations (from 1 to 9) replacing dTs in the following sequence: 5′-TTA CCA TAG AAT CAT GTG CCA TAC ATC ATC-3′. At the 5′-end, a control 25mer is synthesized (QC25), serving as target for the hybridization to its 3′-Cy3-labelled complementary oligonucleotide (QC25c). The cleavage process was monitored by recording the hybridization-based fluorescence intensity before and after the UDG-mediated cleavage of uracil nucleotides. (**B**) Small excerpt (ca. 7% of the total synthesis area) of fluorescence scans before and after enzyme exposure. The scans show the fluorescence intensity, resulting from hybridization to a labelled, complementary oligonucleotide. The microarrays were scanned at 5 µm resolution. (**C**) Decrease in fluorescence intensity for the UDG-mediated uracil excision (thus generating abasic sites) as a function of the number of dU nucleotide incorporations per DNA substrate. The actual cleavage efficiencies correlate with the loss of fluorescence intensity resulting from DNA substrate cleavage. The array was incubated for one hour with UDG and the generated abasic sites were subsequently cleaved under alkaline conditions. The decrease in fluorescence intensity was recorded and normalized to the control strand (U0). The normalized intensities, indicated in arbitrary units, were plotted over the number of dUs per DNA substrate. Error bars are SD.

DNA strand cleavage mediated by UDG consists of two separate steps: excision of the uracil base followed by cleavage of the resulting abasic site. The two steps can be separately monitored on DNA microarrays. First, abasic sites are generated by treating the array with UDG and in a second step, strand cleavage is induced by exposing the surface-tethered, DNA oligonucleotides containing abasic sites to either acidic or basic conditions, or by evaporating the surface to dryness. Such chemical strategies to cleave abasic sites on DNA strands eliminate the need for additional enzymatic cleavage and allow for UDG kinetics to be studied independently. For this purpose, arrays of 30mer DNA sequences with an increasing number of non-consecutive dU-incorporations (dU_1_ – dU_9_) were designed and synthesized by maskless nucleic acid photolithography, using the corresponding photosensitive dU phosphoramidite (Fig. 2). Every DNA strand was synthesized with a dT_15_-linker to the glass surface and terminated at the 5′-end with a 25mer target sequence for hybridization (QC25), as indicated in Fig. 3.

The location of all probe sequences together with corresponding replicates and control strands carrying dT instead of dU nucleotides was randomized across the microarray surface. Prior to enzymatic processing, the DNA probe sequences were hybridized to the Cy3-labelled complementary 25mer oligonucleotide (QC25c) in order to determine initial fluorescence intensity values. 3′-Cy3 labelling was used to prevent possible fluorescence artifacts due to interactions of the dye with the variable number of dUs^46^. The microarrays were then exposed to commercially-sourced UDG from *E. coli* and the abasic site cleavage was subsequently carried out under various conditions. The optimum enzyme exposure time was determined in initial experiments. Our results (Fig. S1) show that after addition of the enzyme, the cleavage efficiency increases significantly with time, up to one hour, reaching an efficiency of roughly 50–60%, with only slight changes upon further exposure to UDG. We thus set the optimum exposure time to one hour. In addition, our data clearly shows increased cleavage efficiency with an increasing number of dU, from 40 to 60% (Figs. 3C and S1).

**Figure 4:**
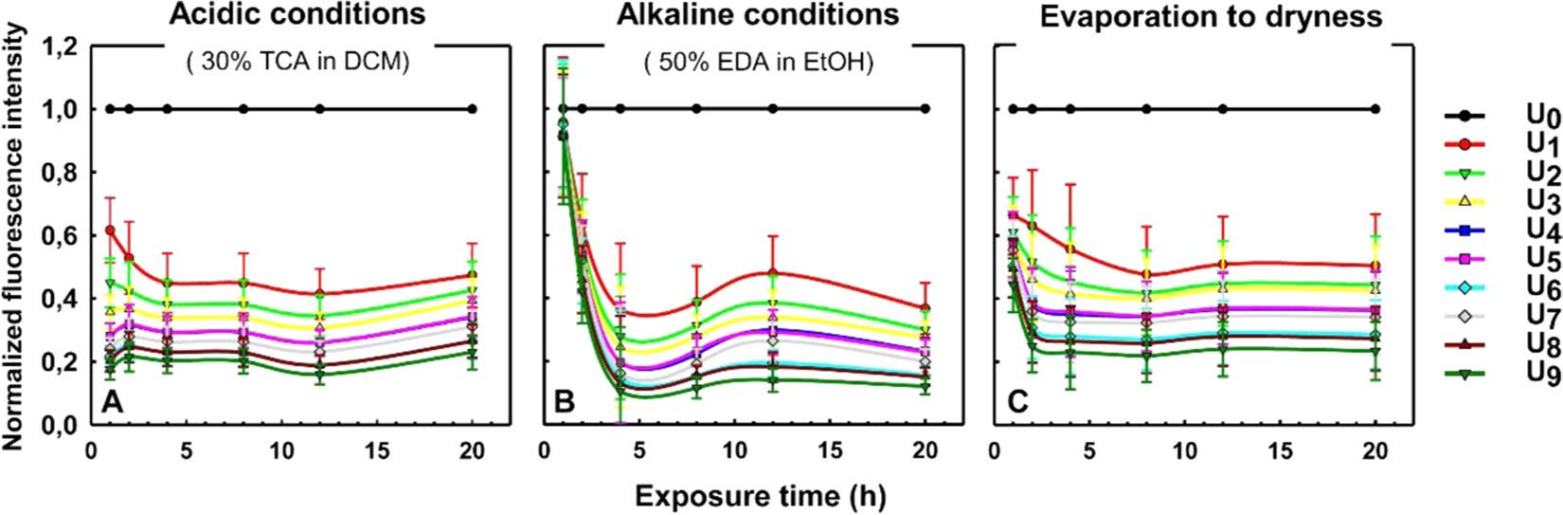
Decrease in fluorescence intensity after chemically-induced abasic site cleavage on substrates with varying number of dU incorporations and as a function of time. The actual cleavage efficiencies correlate with the loss of fluorescence intensity, resulting from DNA substrate cleavage. The decrease in fluorescence intensity was recorded and normalized to that of the control strand (U_0_). The normalized intensities, indicated in arbitrary units, were plotted over the exposure time for the chemical treatments. The DNA array was treated under acidic conditions (**A**), alkaline conditions (**B**) or by evaporating the surface to dryness (**C**) and for various exposure times (1, 2, 4, 8, 12 and 20 hours). The substrate strands contained different ratios of dU nucleotides indicated with different colors: U_0_ (black), U_1_ (red), U_2_ (light green), U_3_ (yellow), U_4_ (blue), U_5_ (pink), U_6_ (turquoise), U_7_ (gray), U_8_ (dark red) and U_9_ (dark green). Error bars are SD.

We then moved on to study the actual cleavage step, the removal of the abasic site after excision of the uracil base. Following known strategies for chemically-induced abasic site cleavage in solution^20,30^, the resulting AP-sites were then cleaved either under alkaline conditions by immersing the arrays into a 1:1 (v/v) solution of ethylenediamine(EDA)/ethanol, or the arrays were treated under acidic conditions by immersing into a 30% (v/v) solution of trichloroacetic acid in dichloromethane, or by evaporating the surface of the array to dryness. In all methods, the treatments were performed for different time periods, ranging from 1 to 24 hours. Subsequently, the arrays were rehybridized to the Cy3-labelled complementary 25mer oligonucleotide (QC25c) and the resulting fluorescence intensities were compared to those obtained before UDG treatment in order to determine cleavage efficiency. The resulting cleavage rates are indicated in Fig. 4.

Under basic conditions (Fig. 4B), the cleavage of AP sites requires a minimum incubation period of 2 hours in order to observe significant cleavage (40 to 60%) of the substrates. However, treating the array with an acid or drying its surface (Fig. 4A,C, respectively) results in clear and extensive cleavage after only one hour (30 to almost 80% with an acidic treatment), when EDA-mediated abasic site cleavage is minimal after one hour. It is likely that the cleavage reaction itself in A and C methods is promoted at the final hybridization stage, either due to temperature or to the presence of an amine in the buffer. Nonetheless, since the hybridization is common to all three methods, the large differences in cleavage kinetics between basic and non-basic treatments hints at the possibility that additional AP sites may be produced in presence of an acid, or under reduced pressure, yielding additional positions on the DNA strands at which a following cleavage reaction can occur. Longer treatments, regardless of the method, do not significantly change the extent of cleavage, usually approaching 40% to 80% depending on the number of dU incorporations. Indeed, we see a clear overall increase in cleavage efficiency with an increasing number of dU-incorporations, under all tested conditions.

In addition to performing chemically induced DNA strand cleavage on abasic site locations, the quality of the microarray surface was examined. For this purpose, the uniformity of the features on the array surfaces was visually inspected based on fluorescence intensities and loss of fluorescence for the non-cleavable control strands was monitored. We found that abasic site cleavage under alkaline conditions allowed for specific, enzyme mediated cleavage of dU-containing sequences but left the control sequences virtually untouched, whereas under acidic conditions, DNA strand cleavage was also observed for control strands lacking uracil nucleotides (Fig. S2). Drying the surface also led to degradation of the dT-only control strands. Since only abasic site cleavage under alkaline conditions avoids surface degradation and non-specific loss of DNA, we performed all subsequent experiments under these conditions. This ensures that the non-cleavable control sequences remain available for comparison even after long chemical treatment.

### Single- and double-stranded UDG sequence dependence

**Figure 5:**
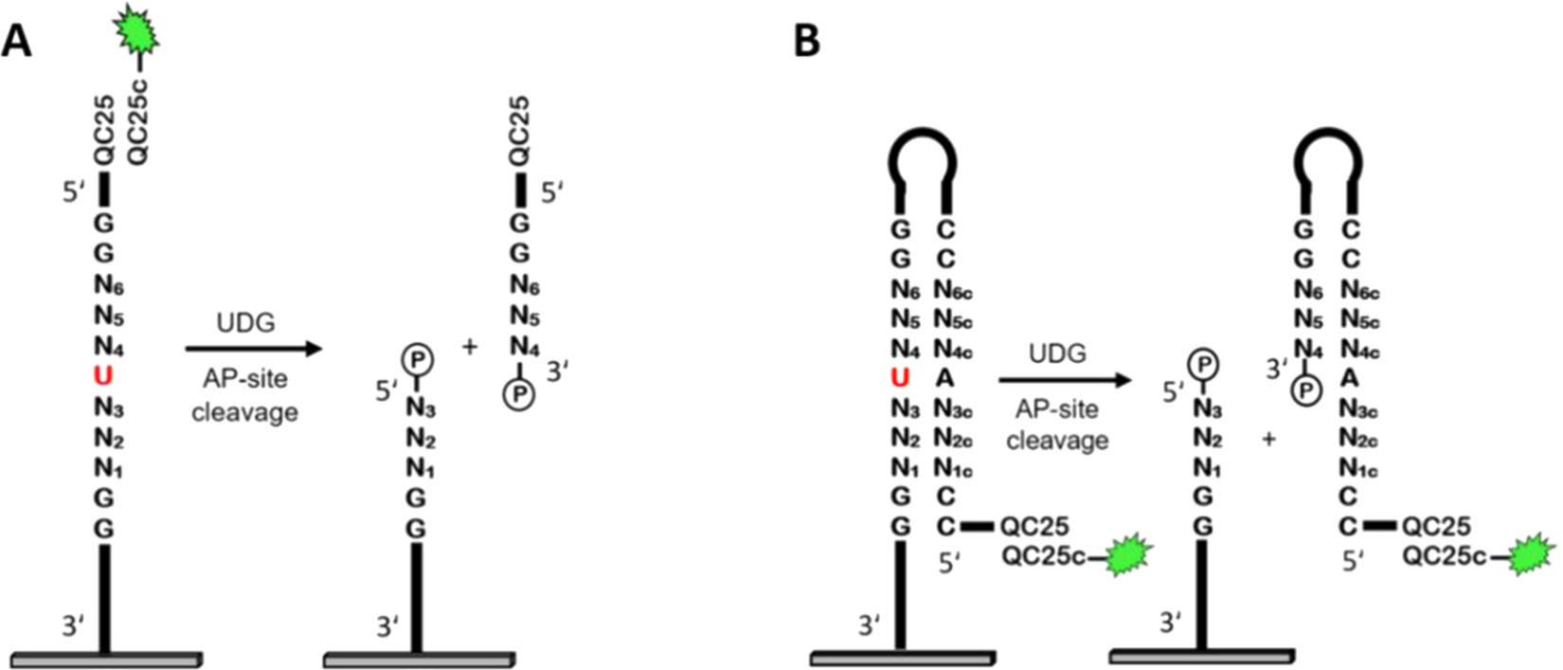
Schematic illustration of the sequence design for the investigation of *E. coli* UDG sequence dependences on single- (**A**) and double-stranded DNA substrates. (**B**) In order to investigate the UDG sequence dependence, a single dU is incorporated into a DNA strand and enclosed by 3 permuted bases on each side. **A** The design for the study of UDG sequence dependence on single-stranded DNA substrates consists of a 15mer dT-linker, a single dU enclosed by 3 permuted bases on each side, followed by a 5′ 25mer sequence (QC25) serving as target for the hybridization to its 3′-Cy3-labelled complementary oligonucleotide (QC25c). **B** For the study of UDG sequence dependence on double-stranded DNA substrates, the sequences were designed to form a hairpin loop. The resulting strands consisted of a 15mer dT-linker, a 11-nt stem, equivalent to the single-stranded design, containing the variable region flanked by dG·dC base pairs, a 4-nt loop followed by the complementary 11nt strand. At the 5′ end, a hybridizable 25mer target sequence (QC25) was synthesized.

Since our results on *E. coli* UDG-mediated dU cleavage delivered only moderate cleavage efficiencies for DNA strands containing single dU incorporations (≈40%), we interrogated the sequence specificity of UDG in order to potentially identify a highly-cleaved dU-containing substrate. For this purpose, we designed a dU-containing nucleic acids library from double- and single-stranded DNA sequences. The library consists of a 7mer permutable sequence with a single dU in the middle (Fig. 5). Permutation of the 3-nt flanking regions, 5′- as well as 3′ of the dU results in 4096 unique sequences. In the double-stranded format, a loop and the complementary sequence to the 7mer were added, leading to the formation of a hairpin structure. Additional dG·dC base pairs in the hairpin stem helped in increasing the melting temperature of the hairpin DNA structure. These base pairs were added to the single-stranded substrates in order to keep the ssDNA and dsDNA designs as similar as possible. Finally, a 25mer oligonucleotide was added to the 5′-end of each design in order to serve as a hybridization target. A representative figure of the sequence designs is shown in Fig. 5.

**Figure 6:**
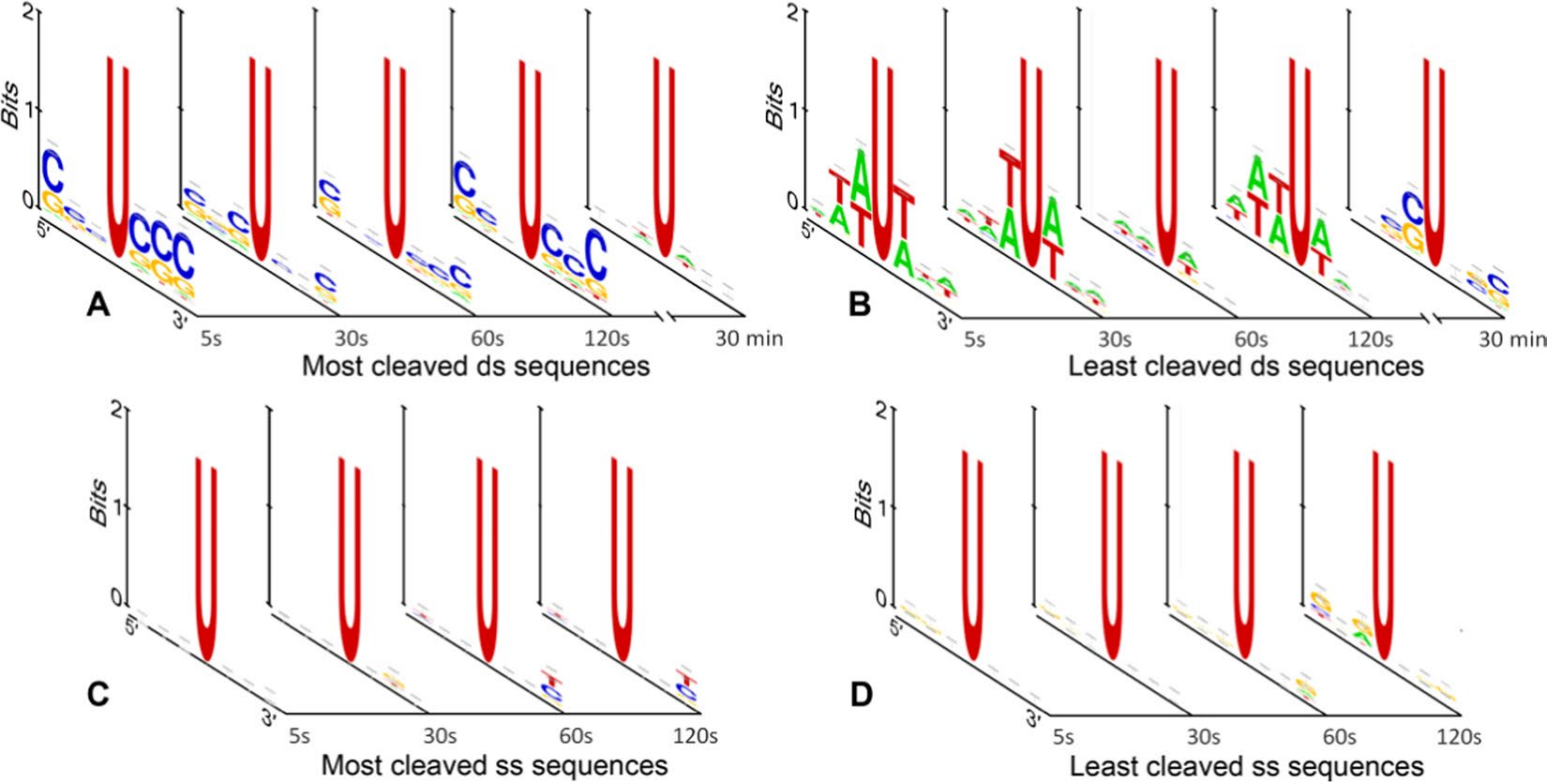
Representative sequence motifs for the UDG-mediated uracil cleavage on double- (**A**,**B**) and single-stranded (**C**,**D**) DNA strands. Substrates were incubated with UDG for different time periods ranging from 5 seconds to 30 minutes (since the cleavage motifs showed little to no sequence dependence, only the 5s, 30s, 60s and 120s were determined for ssDNA). The sequence motifs were extracted from the 1% (41 of 4096 sequences) most cleaved (**A**,**C**) and least cleaved (**B**,**D**) sequences of the library.

Although terminal labeling of DNA with a fluorescent dye is a convenient method to directly measure fluorescence intensity, it carries the disadvantage of high fluorescence noise which, especially on high-density microarrays, makes data extraction and interpretation more difficult. Therefore, we decided to monitor the cleavage reaction via hybridization to the immobilized sequences. Prior to treatment with UDG, the single- and double-stranded DNA substrates were hybridized to the labelled complementary 25mer oligonucleotide and scanned to obtain initial fluorescence values. Then, the microarrays were exposed to UDG for different time periods. After the following AP-site cleavage under alkaline conditions, the arrays were again hybridized to their Cy3-labelled complements. The decrease of fluorescence signal of the DNA strands relative to pre-treatment fluorescence values corresponds directly to cleavage efficiency. Maximum cleavage efficiencies of around 40% were obtained for double-stranded substrates after UDG incubation for 2 minutes, and slightly lower cleavage rates for single-stranded substrates (Table S1). This lower rate for single-stranded contradicts earlier studies indicating that that they are cleaved faster than their double-stranded equivalents^13,47,48^. Interestingly, very short enzyme incubations (<1 min) still lead to significant cleavage efficiencies (30–35%). These short time points are especially interesting since they give clear hints of potential sequence preferences. In order to identify sequence dependence in the UDG-mediated DNA degradation from our set of 4096 individual strands, we focused on the 1% (Fig. 6) as well as 5% (Fig. S3) of the most and least cleaved subset of sequences. Representative sequence motifs generated from those sequence subsets are shown in Fig. 6 and in the Supplementary Information.

For double-stranded DNA (Fig. 6A,B), the results show a clear UDG sequence preference for G/C-rich regions flanking uracil. Conversely, UDG seems to poorly process double-stranded substrates containing T·A base pairs. The motifs extracted from better and poorer UDG substrates are in partial accordance with the very limited existing literature data^48–50^. Seibert *et al*. hypothesized that the efficiency of *E. coli* UDG is related to the energetic cost of DNA bending and distortion occurring in the process of specific damage recognition. In molecular dynamics simulations and time-resolved fluorescence experiments with two uracil-containing double-stranded DNA sequences, they identified that the effective bending force constants are lower if dAs or dTs are located adjacent to the uracil nucleotide, instead of dGs and dCs; suggesting that dA/dT-rich sequences might be more easily bent by UDG and thus better processed^49^. Since single-stranded DNA is a much more flexible form of DNA, its faster processing reported in the literature might be due to the lower energetic cost of cleaving substrates that are inherently more flexible than dsDNA^7,47,49,51^. Bending may not, however, be a deciding factor in UDG cleavage specificity of ssDNA, with the enzyme recognizing and cleaving all substrates equally well, which perhaps explains the absence of sequence consensus in dU-containing ssDNA. It was nevertheless hypothesized that sequence context effects still extend to single-stranded DNA, and indeed had been observed for UDG from herpes simplex virus type 1^52^, but still remains unclear for human or *E. coli* UDG. Slupphaug *et al*. measured the sequence specificity of Human UDG in 34 dsDNA sequence contexts and surmised that dT 3′ to dU always results in slow removal as did, somewhat discordantly, a high GC content^50^. Eftedal *et al*. evaluated the cleavage efficiency of both calf thymus and *E. coli* UDG from 41 dsDNA sequences and found a similar pattern. With our vastly larger set of sequences, we can concur that dT 3′ to dU always results in slow removal, but our data clearly shows that dC, and dG to a lesser extent, 3′ to dU results in fast removal. We were not able to identify significant sequence dependence for dU excision on ssDNA (Fig. 6C,D). It should be noted that the sequence dependence in dsDNA substrates is clear for short enzymatic treatments (5s, 30s, 60s, and 120s), but slowly fades away for longer UDG exposure (30min), indicating that all substrates are eventually cleaved when exposed to the enzyme.

### Cleavage site optimizations

**Figure 7:**
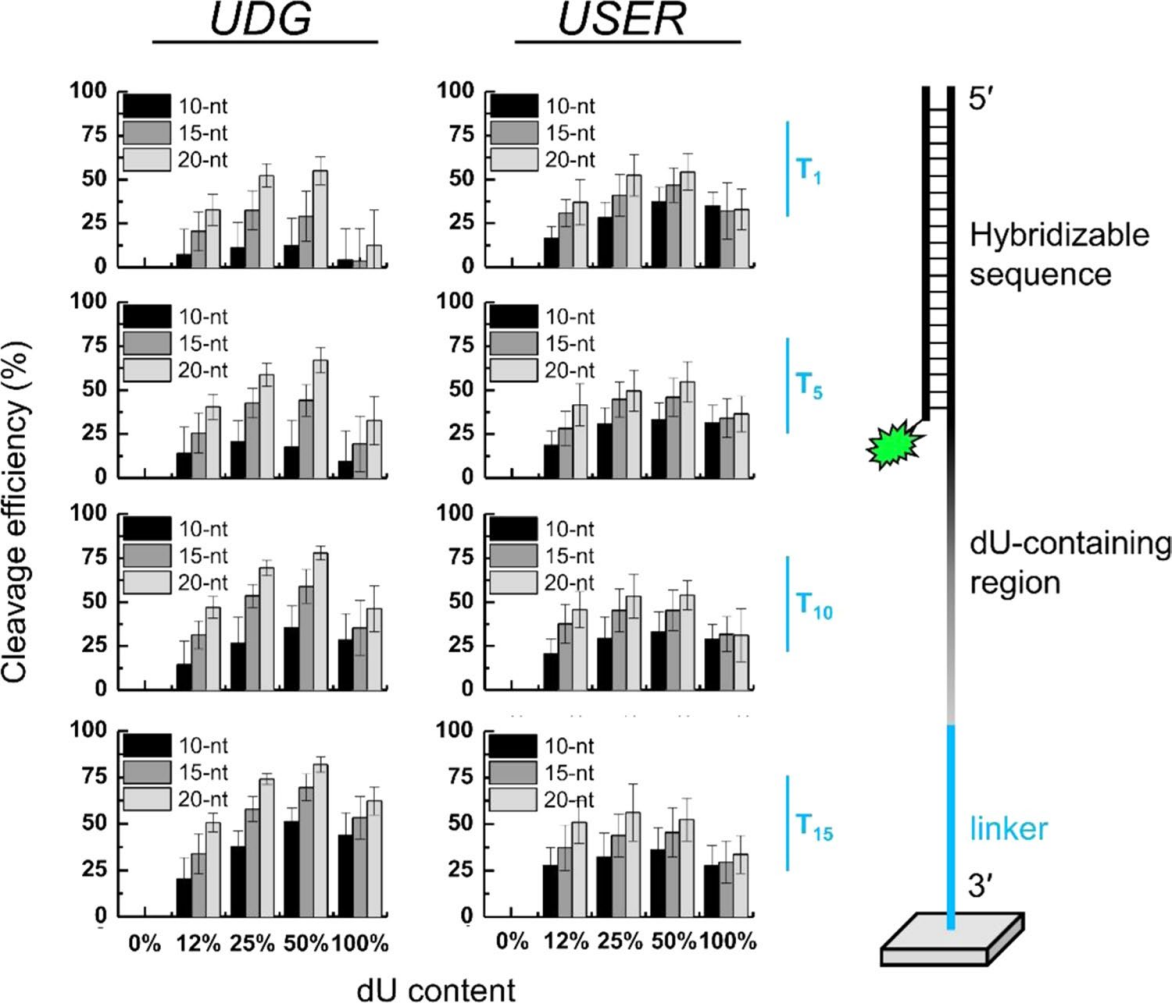
The cleavage efficiency of single-stranded substrates with UDG or USER enzymes. Three parameters are being investigated: linker length (in blue), dU-containing segment length (10, 15 or 20-nt long in black, grey and light grey, respectively) and dU content (from 0 to 100%, on the x axis). All substrates are terminated at the 5ʹ end with a 25mer hybridizable sequence. The corresponding microarrays were treated with either UDG or USER enzyme (5 U, 1 h at 37 °C) and subsequently, in the case of UDG treatment, with EDA/EtOH for 2 h at r.t. The cleavage efficiency corresponds to the loss of hybridization fluorescence after enzymatic treatment and relative to 0%dU controls.

Since no significant sequence dependence for the UDG-mediated uracil excision on single-stranded DNA emerged from our studies and rather moderate cleavage efficiencies for single dU incorporations were obtained, we chose to study UDG-mediated dU excision on longer single-stranded substrates containing various forms of multiple dU incorporations, aiming for identifying specific cleavage sites that are easily enzymatically accessible and allow for fast and efficient strand cleavage. Our rationale is that increasing the distance of the probe from the surface should provide greater accessibility for cleavage, but this effect may not carry over to long spacers (>20-nt)^53^. Based on the results from Fig. 3, we also expect a higher number of dU incorporations to lead to greater overall cleavage, but we wonder if there is an optimal density of dU nucleotides within the cleavable section of the DNA strand. Therefore, we investigated uracil cleavage on longer dU homopolymers (10, 15 and 20mers) as well as on oligomers with varying %dU composition. The percentage of dU within the oligomer was calculated relative to the other four nucleotides (dA, dC, dG and dT) and experimentally achieved by carrying out coupling reactions with pre-mixed solutions of dU/dA/dC/dG/dT phosphoramidites in various ratios (0:100, 12:88, 25:75, 50:50 or 100:0, (v/v) dU:dA/dC/dG/dT). The sequences, built on poly-dT linkers of varying lengths (dT_1_, dT_5_, dT_10_ and dT_15_), and the dU-containing region were then terminated with a 25mer target sequence for hybridization purposes as indicated in Fig. 7.

After synthesis, the 25mer was hybridized to its complementary 5′-Cy3 labelled oligonucleotide and the microarray was exposed to UDG for 1 hour. Then, cleavage of the abasic site was performed under alkaline conditions, followed by a final rehybridization. In parallel, we also performed the cleavage assay with the USER enzyme, which in this case eliminates the need for an additional abasic site cleavage procedure.

For the enzyme assay with UDG followed by abasic site cleavage under alkaline conditions (Fig. 7, left), we recorded cleavage efficiencies ranging from 4% up to 80% and with clear trends emerging. For instance, the cleavage efficiency increases significantly with increasing length of the dU-containing region, in the order 10mer > 15mer > 20mer, with a maximum of ~50% cleavage reachable for a 10mer dU region, and 80% maximum for the 20mer dU region. Similarly, longer linkers results in an overall higher cleavage rate. These observations indicate that longer sequences are better recognized and the corresponding uracil bases better excised by UDG, which in turn suggests a greater accessibility of the longer substrates by the enzyme. The cleavage efficiency is also affected by the amount of dU nucleotides within the cleavable part of the substrate. Indeed, uracil homopolymers (100%dU) are almost always less cleaved than their congeners with interspersed dU nucleotides, and this effect is particularly clear when the substrates are synthesized over a short T_1_ linker. On the other hand, increasing the dU content from 12 to 50% is met with increasing cleavage efficiency, with 50% dU appearing to be the optimal amount. Thus, under our experimental conditions, UDG-mediated cleavage was the most efficient on the longest substrates where half of the nucleotides in the cleavable part are dU (80% cleavage efficiency). This trend, as well as data shown in Fig. 3, suggests that UDG can recognize and bind to multiple dU incorporations, as long as dUs are separated by canonical DNA nucleotides.

While short homopolymers are in general known for being poor UDG substrates, we also expected to observe low cleavage rates for the 20mer cleavable regions since such substrates are unlikely to be found in DNA. *In vivo*, uracil bases mainly occur in DNA strands due to misincorporation during replication or due to deamination^8,9^ and both processes are unlikely to occur at such a high frequency to form homopolymers. Nevertheless, moderate to high cleavage efficiencies of around 60% on long 20mer dU homopolymers were obtained, but whether those homopolymers are processed slower than substrates with lower %dU content remains to be investigated.

Treating a similar DNA array library with the USER enzyme system (Fig. 7, right) leads to satisfactory cleavage of single-stranded substrates, with cleavage trends somewhat comparable to the UDG case but with a few notable exceptions. First, the highest cleavage efficiency is lower than for the UDG/EDA system (55% versus 80%, respectively), which may be attributed to a slower processing of the abasic sites with Endonuclease VIII compared to a common basic treatment with EDA. Second, there seems to be a weaker length discrimination in the cleavable region compared to UDG/EDA, with only 20–25% difference at most between short (10-nt) and long (20-nt) dU-containing regions, when the UDG alone treatment led to large variations in the cleavage efficiency of the same 10 and 20mers (up to 50% difference). Again, this effect could be due to differences in the processing rate of AP sites. Finally, dU homopolymers appear to be degraded to the same extent, regardless of linker or substrate length. This lack of discrimination in the USER enzyme case and compared to UDG-mediated cleavage data suggests that the existence of multiple, consecutive AP sites is the limiting factor in the enzymatic removal of AP sites.

To summarize, in our hands, we found that the best cleavage efficiencies can be achieved with the UDG-mediated cleavage followed by EDA treatment on longer, non-homopolymeric uracil-containing substrates. In so doing, single-stranded sequences containing 50%dU can be efficiently cleaved, albeit in two steps. A short, single-step treatment with the USER enzyme cocktail can also conveniently cleave up to 50% of dU-containing single strands in one hour, however, the apparently lower discriminating power of this particular enzymatic treatment may be less useful when a preferential cleavage is required.

## Conclusion

In this article, we present a large-scale study of the substrate preference and sequence specificity of the uracil-DNA glycosylase on high-density DNA microarrays. Our experimental setup allows for the complete process of uracil removal to be investigated in separate stages: first the cleavage of the uracil bases by incubating the surface-bound DNA strands with UDG, and then, in a second step, the chemical degradation of the resulting abasic sites mediated by an acid, a base or under reduced pressure. Alternatively, cleavage of uracil nucleotides can also be performed in a single reaction step by incubating the arrays with USER enzyme. We studied each of these methods for substrates containing various amounts of dU nucleotides. In summary, we saw that the incubation of high-density DNA microarrays with UDG resulted in uracil-excision leading to the formation of apyrimidinic sites. Such abasic sites were most efficiently cleaved under basic conditions without degradation of the microarray surface. Incubation with USER enzyme proved to be a convenient alternative that allows for mild, relatively efficient and significantly faster uracil-containing strand cleavage, the whole cleavage process being indeed accomplished in 1 h, versus 3 h total for the UDG + EDA approach.

Sequence dependence studies revealed that C/G-rich sequences flanking uracil nucleotides (3′ and 5′) on double-stranded DNA substrates led to much higher UDG cleavage rates than T/A adjacent bases. In contrast, UDG displayed no significant sequence preference on single-stranded substrates. Finally, in an attempt to increase and optimize cleavage efficiency, we found that UDG-mediated uracil removal was dependent on uracil composition, with 50%dU appearing optimal and dropping sharply at higher %dU content. Longer single-stranded substrates were also better recognized and cleaved by UDG or the USER enzyme. Our studies help in further understanding the intrinsic mechanisms of uracil deglycosylation and help in defining the necessary structural requirements for an efficient enzymatic reaction. These efforts will serve to design and create optimal sequence contexts for UDG-mediated DNA degradation, which will prove useful for the controlled release of DNA, RNA and other nucleic acid libraries synthesized on high density microarrays^54–56^.

Due to the fact that UDG is a DNA-specific enzyme that does not excise uracil from RNA substrates, targeted incorporation of deoxyuridine nucleotides into oligonucleotides may, in addition, be used for the specific cleavage of DNA-sections or DNA-templates from DNA:RNA-hybrids. With *in situ* transcription of DNA arrays into RNA, UDG-mediated degradation would be an excellent method to isolate pure, enzymatically-obtained RNA microarrays.

## Methods

### Microarray synthesis

All microarrays were *in situ* synthesized as mirror image pairs via photolithography according to a method published elsewhere^57–59^. Prior to the actual microarray *in situ* synthesis, microscope glass slides (Schott Nexterion Glass D (75 × 25 × 1mm)) that serve as array substrates, were first silanized with *N*-(3-triethoxysilylpropyl)-4-hydroxybutyramide (abcr) in a 95:5 (v/v) solution of ethanol/water containing 0.2% acetic acid for 4 hours^60^. The approach of Maskless Array Synthesis (MAS)^39^ was then used for the light-directed *in situ* fabrication of microarrays, by combining a chemical delivery system and a computer-controlled optical system. For the microarray synthesis, either NPPOC (purchased from ChemGenes) or BzNPPOC^61^ (purchased from Orgentis) DNA phosphoramidites were diluted to 30 mM in acetonitrile (ACN). While the chemical fluidics system (Expedite 8909 nucleic acid synthesizer, PerSeptive Biosystems) provides reagents and solvents, the synchronized computer-controlled optical system serves to pattern UV light via a digital micromirror device (Texas Instruments 0.7 XGA DMD) with 1024 × 768 individually addressable mirrors onto the synthesis area. A U365 surface-mounted UV-LED (Nichia NVSU333A)^62^ served as light source that triggers the removal of the photolabile phosphoramidite protecting groups at the 5′ end. After the synthesis, the microarrays were exposed to a 1:1 (v/v) solution of ethylenediamine/ethanol for 2 hours in order to remove the phosphodiester and nucleobase protecting groups. Subsequently, they were rinsed with deionized water, dried in a microarray centrifuge and stored in a desiccator until further proceeding.

### Microarray hybridization

All DNA microarrays consisted of a linker, an experiment specific section and a 25mer sequence (QC25) that serves as a target for the following hybridization to its complementary DNA oligonucleotide (QC25c) bearing a Cy3 dye at the 5′ or 3′ end.

The QC25 sequence reads as follows:

3′- GTC ATC ATC ATG AAC CAC CCT GGT C-5′

The deprotected microarrays were hybridized as described by Sack *et al*.^57,58^. Briefly, the hybridization was performed in a self-adhesive chamber (Grace Biolabs SA200) by incubating the arrays for 2 hours at 42 °C in a hybridization oven (Boekel Scientific) with a mix consisting of 1× MES, acetylated BSA (10 mg/ml) and 10 nM labelled complementary oligonucleotide. Subsequently, the microarrays were washed in non-stringent wash buffer NSWB (SSPE; 0.9 M NaCl, 0.06 M phosphate, 6 mM EDTA, 0.01% Tween20) for 2 minutes, in stringent wash buffer SWB (100 mM MES, 0.1 M NaCl, 0.01% Tween20) for 1 minute, and in final wash buffer FWB (0.1× SSC) for a few seconds, after which they were dried in a microarray centrifuge.

### Enzyme exposure and abasic site cleavage

Microarrays consisting of 10 different 30mer sequences with increasing numbers of dU incorporations (dU_1_–dU_10_) were designed as shown in Fig. 3. Each sequence consisted of a T_15_-Linker and a random 30mer with an increasing number of dU incorporations, replacing initial dTs, whereas the sequence without any dU incorporation (dU_0_) served as control strand. All strands were terminated with a target 25mer sequence (QC25). The corresponding sequences are shown below:

Surface-[3′]-T_15_-CTA CTA CAT ACC GTG TAC TAA GAT ACC ATT-QC25-[5′] dU_0_ (Control)

Surface-[3′]-T_15_-C**U**A CTA CAT ACC GTG TAC TAA GAT ACC ATT-QC25-[5′] dU_1_

Surface-[3′]-T_15_-C**U**A C**U**A CAT ACC GTG TAC TAA GAT ACC ATT-QC25-[5′] dU_2_

Surface-[3′]-T_15_-C**U**A C**U**A CA**U** ACC GTG TAC TAA GAT ACC ATT-QC25-[5′] dU_3_

Surface-[3′]-T_15_-C**U**A C**U**A CA**U** ACC G**U**G TAC TAA GAT ACC ATT-QC25-[5′] dU_4_

Surface-[3′]-T_15_-C**U**A C**U**A CA**U** ACC G**U**G **U**AC TAA GAT ACC ATT-QC25-[5′] dU_5_

Surface-[3′]-T_15_-C**U**A C**U**A CA**U** ACC G**U**G **U**AC **U**AA GAT ACC ATT-QC25-[5′] dU_6_

Surface-[3′]-T_15_-C**U**A C**U**A CA**U** ACC G**U**G **U**AC **U**AA GA**U** ACC ATT-QC25-[5′] dU_7_

Surface-[3′]-T_15_-C**U**A C**U**A CA**U** ACC G**U**G **U**AC **U**AA GA**U** ACC A**U**T-QC25-[5′] dU_8_

Surface-[3′]-T_15_-C**U**A C**U**A CA**U** ACC G**U**G **U**AC **U**AA GA**U** ACC A**UU**-QC25-[5′] dU_9_

The location of all probe sequences and their corresponding replicates was randomized across the microarray surface. Subsequent to the synthesis, the microarrays were hybridized to a 3′-Cy3-labelled complementary oligonucleotides (QC25c). After enzyme exposure, abasic site cleavage was carried out under various conditions and the microarrays were again hybridized to the 3′-Cy3-labelled QC25c oligonucleotide.

#### Enzyme exposure

Microarrays were incubated with 1× UDG Reaction Buffer (20 mM Tris-HCl, 1 mM DTT and 1 mM EDTA pH 8) and 5 units of UDG (*E. coli* UDG, New England Biolabs, M0280S) in a 300 μl final volume (final enzyme concentration 0.016 U/μl) for either 1 hour or for different time periods ranging from 7 to 120 minutes (7, 15, 30, 60 and 120 min) at 37 °C in a hybridization oven (Boekel Scientific). Subsequently, the microarrays were rinsed in deionized water and dried in a microarray centrifuge.

#### Alkaline conditions

Microarrays were immersed into a 1:1 (v/v) solution of EDA/EtOH for either 2 hours or for different time periods, ranging from 1 to 24 hours (1, 2, 4, 8, 12 and 24 h). They were subsequently washed in deionized water and dried in a microarray centrifuge.

#### Acidic conditions

Microarrays were immersed into a 30% solution of TCA in DCM for different time periods, ranging from 1 to 24 hours (1, 2, 4, 8, 12 and 24 h). They were subsequently washed in deionized water and dried in a microarray centrifuge.

#### Evaporation to dryness

Microarrays were evaporated to dryness in a vacuum oven for different time periods, ranging from 1 to 24 hours (1, 2, 4, 8, 12 and 24 h). They were subsequently washed in deionized water and dried in a microarray centrifuge.

### Sequence dependence experiments

#### Double-stranded substrate

Microarrays were *in situ* synthesized with sequences designed to form a hairpin loop. The resulting strands consisted of a 15mer dT-linker, a 11-nt stem containing the variable region framed by dG nucleotides so as to increase the melting temperature of the hairpin, a 4-nt loop followed by the other branch of the stem. The strand design was terminated with a 5′ 25mer target sequence (QC25) for hybridization. The 7-nt variable region consisted of a single dU enclosed by 3 permuted bases on each side as shown below:

Surface-[3′]-T_15_-GG-N_1_N_2_N_3_-**U**-N_4_N_5_N_6_-GG-C-T-T-C-CC-N_6c_N_5c_N_4c_-A-N_3c_N_2c_N_1c_-CC-QC25-[5′]

For 4 given bases (A, C, G, T) the 6 permutation locations result in 4096 possible permuted sequences. Out of those, 10 sequences were randomly chosen and modified by replacing the dU with dT in order to serve as control strands. The location of all probe sequences together with their corresponding replicates was randomized across the microarray surface.

#### Single-stranded substrate

Microarrays were *in situ* synthesized with sequences consisting of a 15mer dT-linker, a single dU enclosed by 3 permuted bases on each side, followed by a 25mer sequence (QC25) as shown below:

Surface-[3′]-T_15_-GG-N_1_N_2_N_3_-**U**-N_5_N_6_N_7_-GG-QC25-[5′]

Additionally, two dG nucleotides were incorporated before and after the permuted region in order to resemble the double-stranded template as much as possible. Again, 10 sequences out of the 4096 possible permuted sequences were chosen and the dU swapped with dT in order to serve as control strands. The location of all probe sequences together with their corresponding replicates was randomized across the microarray surface.

The *in situ* synthesized microarrays were incubated with 1× MES hybridization buffer for 5 minutes at 65 °C and cooled down to 25 °C over a period of 1 hour. Then, the 3′-Cy3-labelled QC25c oligonucleotide was added to the hybridization mix and the arrays were incubated for another hour at 25 °C. They were then exposed to UDG (5 U, 37 °C) in UDG buffer (20 mM Tris-HCl, 1 mM DTT and 1 mM EDTA pH 8) in a 300 μl final volume (final enzyme concentration 0.016 U/μl) for various time periods ranging from 5 seconds to 30 min and the resulting abasic sites were cleaved under alkaline conditions (EDA/EtOH, 1:1 (v/v %)) for 2 hours at room temperature. After washing the arrays thoroughly with deionized water, they were rehybridized to the 3′-Cy3-labelled QC25c oligonucleotides. All hybridizations and the enzyme exposure together with the abasic site cleavage process were performed as described above.

### Cleavage site optimization

Microarrays carrying sequences with various uracil cleavage sites were synthesized. The strands consisted of a dT linker of varying length (dT_1_, dT_5_, dT_10_ and dT_15_), the uracil cleavage site and a 25mer target sequence (QC25) for further hybridizations. The actual cleavage sites consisted of different lengths ranging from 10 to 20mers (10mer, 15mer and 20mer) as well as of different nucleotide composition (0:100, 12:88, 25:75, 50:50 and 100:0 (v/v %) dU:equimolar mix of dA/dC/dG/dT). The location of all probe sequences and their corresponding replicates was randomized across the microarray surface. After synthesis and deprotection, the microarrays were hybridized to their 5′-Cy3-labelled complementary oligonucleotides (QC25c). Then, the arrays were either exposed solely to USER enzyme (5 U, 1 h, 37 °C, expressed and purified from *E. coli*, New England Biolabs, M5505S) in 1× USER buffer (50 mM KOAc, 20 mM Tris-acetate, 10 mM Mg(OAc)_2_ and 100 µg/ml BSA pH 7.9) or to UDG (5 U, 1 h, 37 °C) followed by abasic site cleavage under alkaline conditions (EDA/EtOH, 1:1 (v/v %), 2 h, r.t). Working enzyme concentration for UDG and USER was 0.016 U/μl in a 300 μl final volume, as described above. After rising the microarrays with deionized water, they were again hybridized to the 5′-Cy3-labelled QC25c oligonucleotide. All hybridizations and the enzyme exposure together with the abasic site cleavage process were performed as described above.

### Data extraction and analysis

In order to obtain fluorescent images, the dried microarrays were scanned at a 5 µm resolution with an excitation wavelength of 532 nm using the microarray scanner GenePix 4100A (Molecular Devices). Data extraction of the scanned images was performed with the software NimbleScan 2.1 (Roche NimbleGen). The fluorescence intensity values of each microarray were calculated as the average of the replicates of each sequence. For the investigation of sequence dependences background values were subtracted from measured values. The cleavage efficiency was then calculated according to the following equations:

$$A=\frac{{I}_{cleavablesequencesbeforeUDG}-{I}_{backgroundbeforeUDG}}{{I}_{controlbeforeUDG}-{I}_{backgroundbeforeUDG}}$$

$$B=\frac{{I}_{cleavablesequencesafterUDG}-{I}_{backgroundafterUDG}}{{I}_{controlsequencesafterUDG}-\,{I}_{backgroundafterUDG}}$$

$$Cleavage\,efficiency=(1-\frac{B}{A})$$

Here *I* represents the measured fluorescence intensity (in arbitrary units). The consensus sequence logos were generated with WebLogo^63^ (https://weblogo.berkeley.edu/) based on the list of 5′ to 3′ sequences, containing the variable region only.

## Supplementary information

Supplementary information
