# Supplementary material for: Specificity and Efficiency of the Uracil DNA Glycosylase-Mediated Strand Cleavage Surveyed on Large Sequence Libraries
Source: Sci Rep. 2019 Nov 28;9:17822. doi: 10.1038/s41598-019-54044-x (PMC6883067; doi:10.1038/s41598-019-54044-x)
Supplement: Supplementary file 1 — Supplementary information [file 41598_2019_54044_MOESM1_ESM.pdf]

Supplementary information (SI)

"Specificity and Efficiency of the Uracil DNA Glycosylase-Mediated Strand Cleavage Surveyed on Large Sequence Libraries"

Kathrin Hölz[1], Angelina Pavlic[1], Jory Lietard[1]* and Mark M. Somoza[1,2]*

Email:

*jory.lietard@univie.ac.at

*mark.somoza@univie.ac.at

[1]Institute of Inorganic Chemistry, Faculty of Chemistry, University of Vienna, Vienna, Austria

[2]Chair of Food Chemistry and Molecular Sensory Science, Technical University of Munich, Lise-Meitner-Straße 34, 85354 Freising, Germany

Table of Contents

1. UDG exposure S2
2. UDG sequence dependence S3
3. List of most-cleaved ss- and dsDNA substrates S4

Uracil-DNA glycosylase exposure

In order to investigate uracil-DNA glycosylase (UDG) mediated uracil excision from uracil-containing DNA strands on microarray surfaces, microarrays consisting of 10 different 30mer sequences with increasing numbers of non-consecutive dU incorporations (dU_1-dU_{10}) were designed as indicated in Figure 3. Each sequence consisted of a T_{15}-Linker and a random 30mer with an increasing number of dU incorporations, replacing initial dTs, whereas the sequence without any dU incorporation (dU_0) served as a control strand. All strands were terminated with a target 25mer sequence (QC25) for hybridization to a Cy3-labelled complementary oligonucleotide (QC25c). The microarrays were incubated with UDG for different time periods ranging from 7 minutes up to 2 hours. After uracil excision, the UDG-generated abasic sites were cleaved chemically by immersing the array into an alkaline solution (EDA/EtOH, 1:1 (v/v)). Subsequently, the array was re-hybridized with a labelled oligonucleotide. The fluorescence intensities were recorded and normalized to that of the control strand. The cleavage efficiency represents the loss of fluorescence intensity after enzymatic exposure.

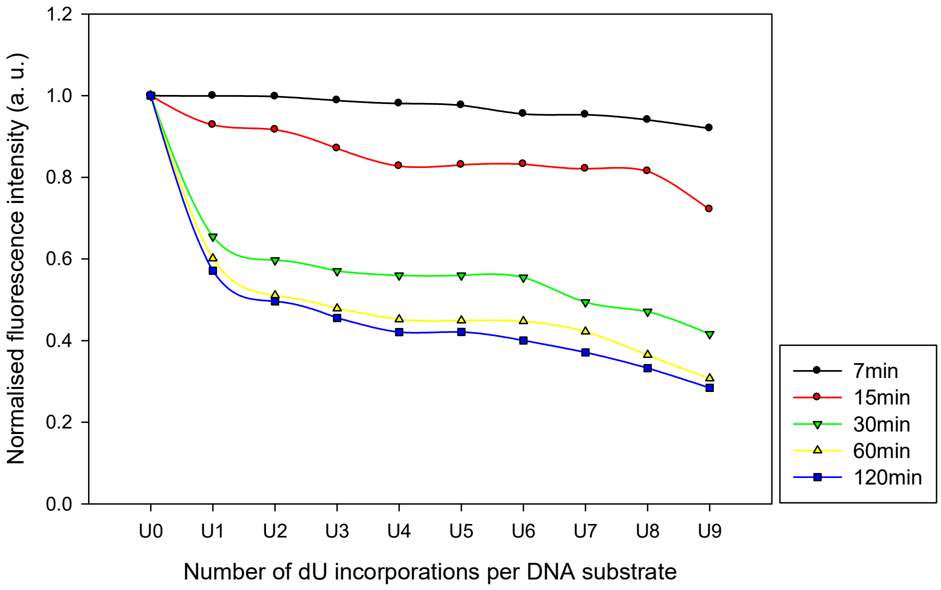

Figure S1. Decrease in fluorescence intensity representing the efficiency of UDG-mediated uracil excision and, hence abasic site generation as a function of the number of dU nucleotide incorporations per DNA substrate. The actual cleavage efficiencies correlate with the loss of fluorescence intensity, resulting from DNA substrate cleavage. The arrays were incubated with UDG and the generated abasic sites were subsequently cleaved under alkaline conditions. The decrease in fluorescence intensity was recorded and normalized to that of the control strand (U_0). The normalized intensities, indicated in arbitrary units, were plotted over the number of dUs per DNA substrate. UDG incubation was performed for various time periods, indicated with different colors: 7min (*black*), 15min (*red*), 30min (*green*), 60min (*yellow*) and 120min (*blue*).

Surface degradation upon contact with an acid or a base, or under reduced pressure

In order to estimate the extent by which oligonucleotides synthesized on a microarray are affected by the various treatments (3% trichloroacetic acid in dichloromethane, ethylenediamine/ethanol 1:1 or vacuum) following exposure to UDG, we monitor the cleavage of a DNA control strand without any dU nucleotide (U0, see also Figure 3A). Loss of absolute fluorescence intensity upon treatment followed by re-hybridization correlates with cleavage/degradation of the control DNA strands. The results are shown in Figure S2. There is rapid degradation (loss of fluorescence upon re-hybridization) of the control oligonucleotides under acidic conditions and significant loss of hybridization efficiency if the array left under reduced pressure. On the other hand, if the microarray is treated with a basic solution following UDG exposure, the control oligonucleotides hybridize to their complement with similar efficiency after 2h in EDA (Figure S2, red curve), when the same amount of time in contact with an acid (black curve) or under vacuum (green curve) leads to important loss of hybridization efficiency. Hybridization of U0 after a 4h treatment with EDA is unusually high but shows that EDA-mediated surface degradation is limited. Control oligonucleotides slowly undergo degradation under prolonged exposure to EDA (> 10h).

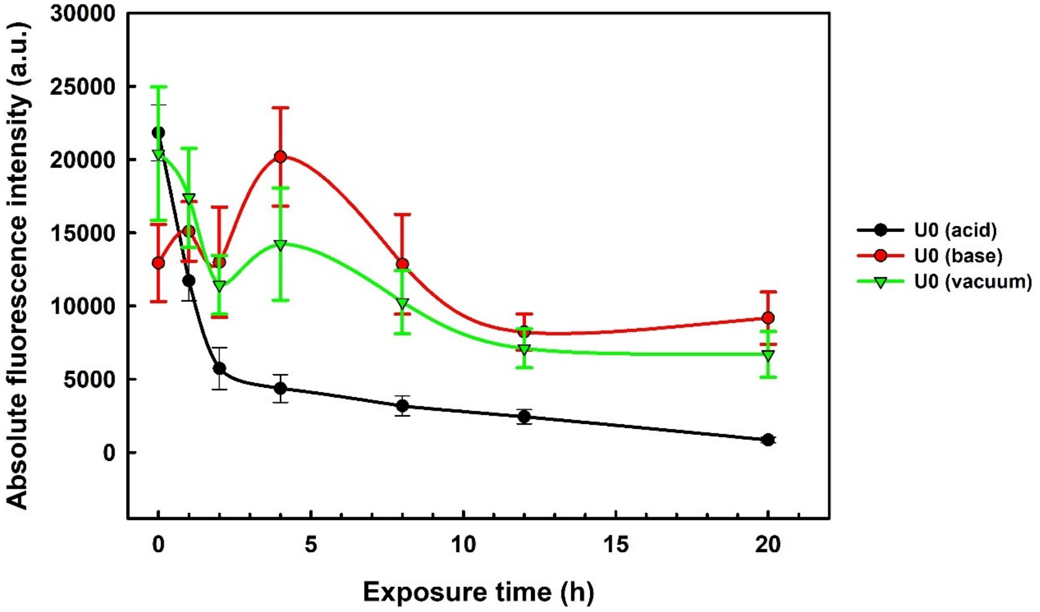

Figure S2 Absolute fluorescence intensities of a control DNA oligonucleotide with dT replacing dU (U0) hybridized to its Cy3-labelled complementary strand after treatment of the AP-site containing microarray with either: 3% trichloroacetic acid in dichloromethane (acid, black curve), ethylenediamine/ethanol 1:1 (base, red curve) or after drying the surface under reduced pressure (vacuum, green curve). Error bars are SD.

UDG Sequence dependence

Representative sequence motifs for the UDG-mediated uracil cleavage on double- and single-stranded DNA strands are shown below. Nucleic acid substrates were incubated with UDG for different time periods ranging from 5 seconds to 30 minutes. The sequence motifs were extracted from the 1% (Figure S3, top) and 5% (Figure S3, bottom) of most cleaved and least cleaved sequences of the library.

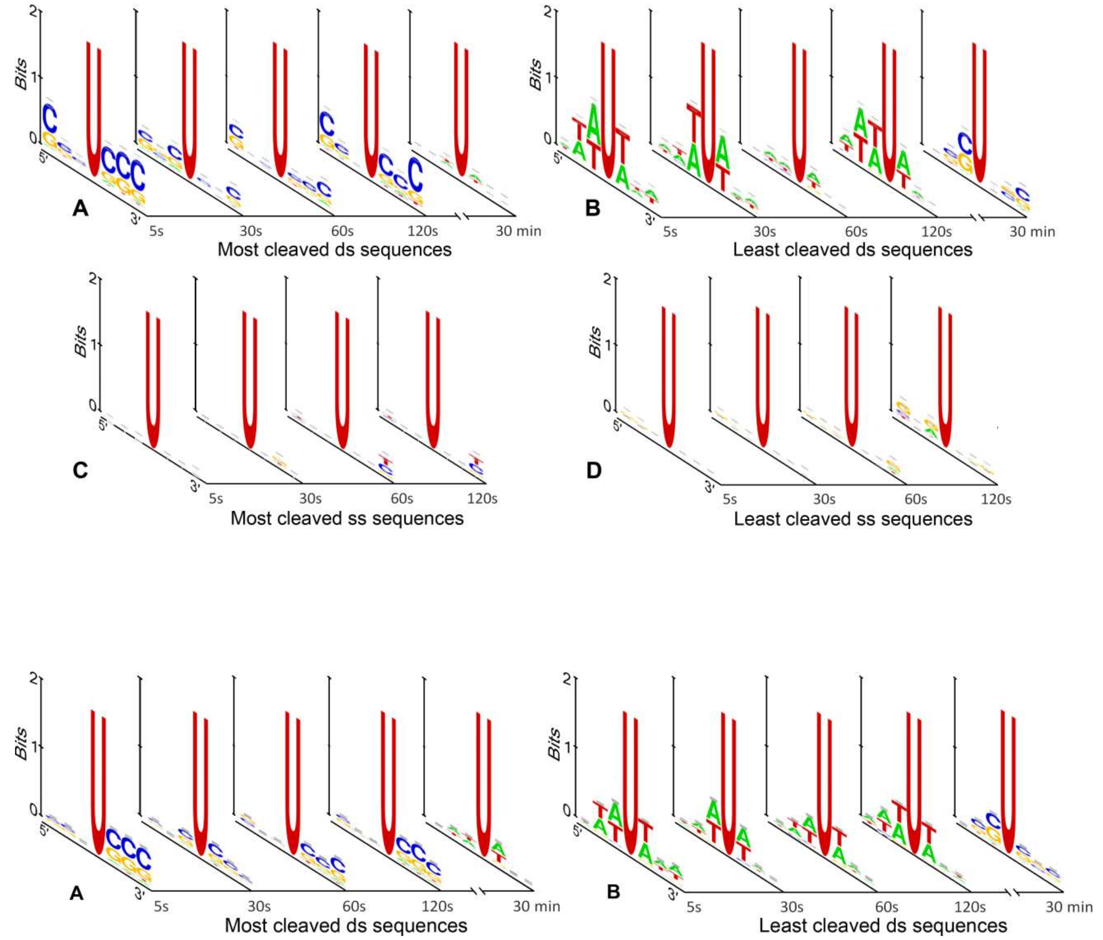

Figure S3 (Top) Representative sequence motifs for the 1% (41 of 4096 sequences) of most cleaved (**A** and **C**) and least cleaved (**B** and **D**) sequences of the library on double-stranded (**A** and **B**) single-stranded (**C** and **D**) DNA substrates. **(Bottom)** Representative sequence motifs for the 5% (205 of 4096 sequences) of most cleaved (**A**) and least cleaved (**B**) double-stranded sequences. The equivalent sequence motifs for the 5% most and least cleaved single-stranded DNA substrates show no sequence motif and are not shown.

Table S1. List of top 100 most-cleaved substrates in single stranded and double-stranded form after a 2 min incubation with UDG followed by abasic site cleavage under basic conditions, with the corresponding calculated cleavage efficiency. Cleavage efficiency is calculated as the loss of fluorescence intensity after enzymatic treatment, and relative to an uncleavable control. The substrate sequence is shown in the 5′ to 3′ order and, in the dsDNA form, corresponds to the sequence of the 3′ branch.

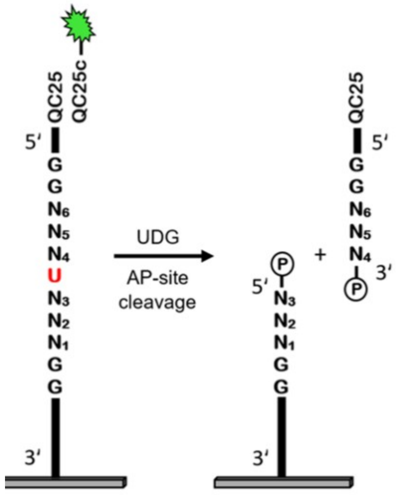

ssDNA

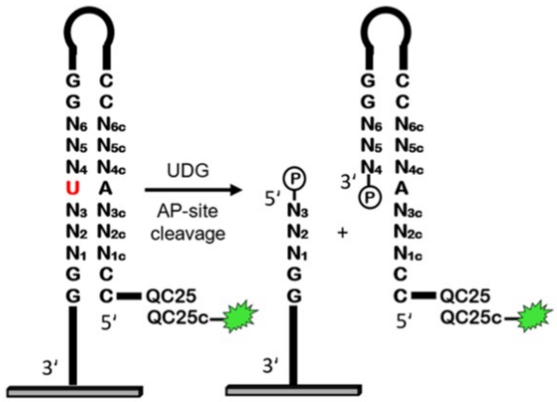

dsDNA

Sequence (5' to 3')	Cleavage efficiency (%)	Sequence (5' to 3')	Cleavage efficiency (%)
CATUTCC	37.3	GAGUGGT	39.1
AAGUATC	37.0	CTTUCCC	39.0
CAAUAAC	35.0	GGAUGAG	38.8
CCAUGGT	32.2	CCCUCCC	38.6
GCCUAAG	32.2	GCCUCCC	38.4
ACTUAAG	31.8	CCGUCCC	37.8
ACAUCCA	31.5	CCTUCCT	37.3
TTTUAAT	30.3	CCCUCAC	37.2
GTGUTGA	29.8	GGGUCGG	36.9
CTGUATC	29.7	CCTUCCC	36.8
GCCUGCA	28.3	GCTUCCC	36.6
CTAUAGT	27.9	CCCUTTC	36.4
AAAUCCG	27.6	CCCUCTC	36.4
AGTUCCT	27.1	CCCUGCC	36.2
TGAUCCG	27.1	CACUCCC	36.0
CCGUGCT	26.9	CGGUCCC	35.6
CTCUGAA	26.9	GGAUGGG	35.6
ATAUGGG	26.5	CCTUTTC	35.5
CTTUCCT	26.4	AGCUCCC	35.4
TTCUATG	26.1	GAAUGGG	35.3
CGTUAGA	26.1	CCTUCAC	35.3
CTAUCAC	26.0	GGGUCAG	35.2
CCCUCCG	25.9	TCCUCCC	35.2
ACCUCCC	25.9	GGGUAGG	35.2

CTCUTTA	25.8	GTGUCCC	35.0
AGCUGCG	25.6	CTCUCCC	34.9
GTAUTCT	25.5	GGTUAAG	34.9
GTCUTTA	25.4	CCAUGCC	34.8
GAGUGTT	25.2	TGAUCCC	34.7
CCTUTAG	25.1	GGGUGGA	34.7
TTCUCGG	24.6	CCAUGGG	34.6
TCTUGCT	24.6	CCTUACC	34.5
CGTUTTT	24.5	CCAUCTC	34.5
CCAUCCC	24.5	ACCUCCC	34.4
GTCUAAC	24.4	CCAUTCC	34.3
TGAUATC	24.4	CATUCCC	34.2
GTTUTTG	24.3	CTCUCTC	34.2
GTGUGCA	24.3	CCCUCCT	34.2
AGAUCTC	24.2	CCGUGCC	33.8
TATUCTC	24.2	CAAUCCC	33.8
GCAUGCT	24.1	CTGUCCC	33.8
AGGUCCT	24.1	GGTUCGG	33.7
GCTUCTT	23.9	CCCUCCA	33.6
GTCUTCT	23.8	CGCUCCC	33.6
ACTUATG	23.8	CCCUTCC	33.6
ACTUAGT	23.8	TGGUCCC	33.5
TTGUGAC	23.7	CTCUGCC	33.5
GAAUTTG	23.7	TGTUCCC	33.4
GTCUCCA	23.7	TGGUAGG	33.4
AATUGTG	23.6	CGGUCGG	33.3
TTTUCTC	23.6	ACCUTCC	33.3
CAAUTGC	23.6	GAGUCGG	33.2
GCCUTCT	23.5	CCCUACC	33.2
TTGUGGG	23.5	CACUTCC	33.2
TAGUTCA	23.3	GACUGGT	33.2
AGCUGAA	23.3	TCAUCCC	33.2
TACUTAA	23.3	ACGUCGG	33.1
AGTUTCT	23.3	CTCUCCA	33.1
AAGUGGT	23.2	GCCUTCC	33.1
ACCUCTC	23.2	TGCUTCC	33.1
ACCUGCC	23.1	GGAUCAG	33.0
TCCUTCC	23.0	TGCUCCC	33.0
CCTUCTC	22.9	AGGUCCC	33.0
TACUGGG	22.9	CTAUCCC	32.9
CCAUCTA	22.8	CGCUGCC	32.9
TCCUTGC	22.8	ACCUCTC	32.9
CTCUATC	22.8	AGGUAGG	32.9
TGTUACA	22.8	GCAUCCC	32.9
ATCUGTT	22.7	GGAUCGG	32.8
TGAUCCA	22.7	GGTUAGG	32.7
TGTUGAC	22.7	CTGUGCC	32.7

GCCUTCC	22.6	CTCUCAC	32.5
GGGUGCA	22.6	GTGUCGG	32.5
GCAUAAA	22.5	GAGUGGA	32.4
CACUGCC	22.5	GGGUCCC	32.3
CGCUAGC	22.5	ATCUCCC	32.3
TCTUAGT	22.4	GGGUGGT	32.2
ATTUTAC	22.4	CCTUCCA	32.2
ATCUTGA	22.4	CGCUTCC	32.1
AATUTTC	22.2	GGTUCAG	32.1
GATUATC	22.1	GACUGAG	32.0
CACUTAC	22.1	CTGUACC	32.0
CCCUTAA	22.1	GAGUCAG	31.9
GAGUCGT	22.0	GACUCGG	31.9
CTAUTTT	22.0	ACAUGGG	31.9
GCCUATC	22.0	CTGUCCA	31.8
CGCUCGC	22.0	TCTUCCC	31.8
AGTUACC	21.9	ACTUCCC	31.7
CTAUGAT	21.9	CGCUCCA	31.7
TACUCCC	21.8	GGCUCCC	31.7
AGTUAAA	21.8	CAGUCCC	31.7
GTCUTAA	21.8	TGGUGGA	31.7
TAGUACT	21.8	AGGUCGG	31.6
CTCUATG	21.8	GCCUCGG	31.6
ACTUCTG	21.8	GATUAGG	31.5
GTTUTGG	21.7	GTGUGGA	31.5
TCTUGTT	21.7	CCGUCGG	31.5
CCTUGAA	21.7	CTCUCCT	31.5
CCTUAGT	21.7	AAAUCCC	31.4
GTGUGCT	21.7	TTTUCCC	31.4
